# Return to Sports in Recreational Athletes After Complex Elbow Dislocation

**DOI:** 10.3390/jcm15051702

**Published:** 2026-02-24

**Authors:** Stephan Regenbogen, Philip-Christian Nolte, Marc Schnetzke, Melina Vorm Walde, Jennifer Bruttel

**Affiliations:** 1 BG Klinik Ludwigshafen, Department for Orthopaedics and Trauma Surgery, Heidelberg University, Ludwig-Guttmann-Str. 13, 67071 Ludwigshafen, Germany; 2 German Joint Centre, ATOS Clinic Heidelberg, Bismarckplatz 9-15, 69115 Heidelberg, Germany; marc.schnetzke@atos.de; 3 University Hospital Heidelberg, Im Neuenheimer Feld, 69120 Heidelberg, Germany; melina.vormwalde@med.uni-heidelberg.de

**Keywords:** complex elbow injury, elbow fracture, return to work, elbow instability, return to sport, elbow stabilization

## Abstract

**Background/Objectives**: Complex elbow dislocations are rare but severe injuries. Optimal treatment is controversial, and few studies focus on return to sports. The aim of the study is to analyze functional outcomes and return-to-sport and return-to-work metrics in recreational athletes after complex elbow dislocation. **Methods**: A retrospective single-center study at a level I trauma center was performed from January 2008 to December 2019. Epidemiological data, associated injuries, and treatment of complex elbow dislocations were analyzed. Patients were followed up for at least two years after injury, and return-to-sports and return-to-work rates along with patient-reported outcome measures were investigated. **Results**: Fifty-six patients were included. The mean follow-up was 6.4 years (range, 2.0–12.5). The mean Subjective Elbow Value was 80.2 (95% CI, 74.7–85.8), the Mayo Elbow Performance Score was 83.8 (95% CI, 78.8–88.7), and Oxford Elbow Score was 37.8 (95% CI, 35.1–40.5). Of 41 recreational athletes, 37 returned to sports; however, only 31.7% fully returned to their pre-injury sport, 43.9% returned partially, and 24.4% switched sports. A total of 82% fully returned to work, 8.2% partially, and 10.2% did not return. **Conclusions**: Complex elbow dislocations remain severe injuries associated with relevant functional limitations. Nevertheless, good clinical outcomes and high overall return-to-sport and return-to-work rates can be achieved in recreational athletes. While the majority resume sporting activity, nearly 25% of patients do not return to their pre-injury sport.

## 1. Introduction

Injuries to the elbow are common during sport activities, especially in adolescent males and young athletes [1,2].

With an incidence of approximately 6 in 100,000 [3], dislocations of the elbow represent the second most common dislocation of the upper extremity [1,4]. About half of these injuries are related to sports [5].

Elbow dislocations are classified as simple if they are without relevant osseous lesions [6]. Complex elbow dislocations are defined as elbow dislocations associated with concomitant fractures that extend beyond simple avulsion fractures of the coronoid process and typically arise from high-energy trauma, such as falls from heights, sports injuries, or motor vehicle accidents, making them particularly prevalent among young, active individuals and athletes [1,2,7]. Accompanying fractures occur in 26% of all elbow dislocations [7,8]. There is an increasing diversity of complex elbow dislocation patterns extending beyond classic classifications such as the terrible triad.

Common injury patterns in complex elbow dislocations are proximal ulna fractures, including Monteggia and Monteggia-like injuries, as well as terrible triad injuries, which are defined as an elbow dislocation with a radial head and coronoid fracture [9,10]. Terrible triad injuries occur in 87% of complex elbow dislocations [11]. Overall coronoid and radial head fractures are the most common osseus injuries in complex elbow dislocation [12]. Often, these fractures are accompanied by ligamentous injuries, most commonly of the lateral collateral ligament [13,14]. Advances in imaging and surgical techniques have revealed heterogeneous combinations of osseous, ligamentous, and soft tissue injuries, underlining the need for injury-specific outcome analyses [6,7,8].

Due to the elbow’s intricate anatomy, comprising the humerus, ulna, radius, and network of ligaments and neurovascular structures, a complex elbow dislocation is a significant orthopedic injury. Therefore, treatment is challenging, and unsatisfactory patient outcomes such as chronic instability and posttraumatic arthrosis have been observed [4,7,15]. As the elbow is not only critical for upper extremity movement but also plays a pivotal role in everyday activities and sports, these complications can significantly impact an individual’s ability to return to work and engage in sports, emphasizing the importance of timely and appropriate treatment.

In general, returning to sports and work without restrictions after major trauma is often impeded by residual disability and disability-adjusted life-years (DALYs) accounted for 248 million US dollars in 2021 [16]. Societal costs of productivity loss might even exceed the costs of medical care [17]. Therefore, returning to sports and to work following a complex elbow dislocation is of paramount importance for many individuals both for their physical health and mental well-being, but it may also have an enormous societal impact. Recent literature showed that conservative and surgical treatment results in high patient satisfaction, good-to-excellent functional outcomes, and a high return-to-sport rate in recreational athletes with simple elbow dislocations [18,19]. However, there is a lack of evidence on return to work and return to sports after complex elbow dislocation. As recently discussed by Cueto et al., there is great heterogeneity in patient-reported outcome measures (PROMs) in the literature; therefore, comparability is limited [11]. Furthermore, due to the broad spectrum of elbow injuries that are classified as “complex dislocation”, there is a high variability in the reported clinical outcomes [11]. Most patients achieve good postoperative functional results with standardized treatment protocols. For instance, the mean Mayo Elbow Performance Score (MEPS) in one prospective study was 88.8 ± 17.6, indicating favorable elbow function for most, although postoperative complications occurred in about 31% of cases, and roughly 25% required surgical revision [20]. Another retrospective study with 44 patients reported similar results after surgical treatment, with a mean MEPS of 86.7 (range 30–100) and a mean Oxford Elbow Score (OES) of 40.4 (range 15–48) [4]. The literature regarding return to work and sports after complex elbow dislocation is almost completely lacking.

Therefore, the aim of the study is to analyze functional outcomes and return-to-sport and return-to-work metrics in recreational athletes who suffered a complex elbow dislocation. It was hypothesized that a complex elbow dislocation would lead to significant restrictions in return to sports and return to work.

## 2. Materials and Methods

### 2.1. Patients and Study Design

This retrospective study examined all consecutive patients who sustained a complex elbow dislocation from January 2008 to December 2019 and were treated operatively at a level I trauma center. The study was approved by the local medical ethics committee (2020–15412), and all participants gave written informed consent before the follow-up.

**Figure 1:**
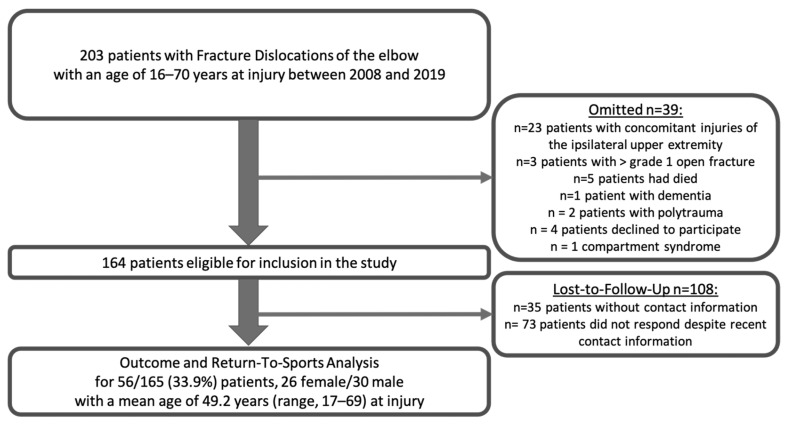
Flowchart illustrating inclusion and exclusion criteria.

The inclusion criteria were as follows: (1) acute (< 3 weeks) complex elbow dislocation, (2) 16–65 years of age, (3) pre-reduction imaging and post-reduction CT scans following injury, and (4) a minimum follow-up of >2 years. The exclusion criteria were as follows: (1) incomplete or missing medical records or (2) missing pre-reduction radiological imaging and post-reduction CT scans, (3) concomitant injuries of the ipsilateral upper extremity, (4) >grade 1 open fracture, (5) previous injuries to the elbow, (6) polytraumatized, (7) mental conditions such as dementia, and (8) compartment syndrome (Figure 1).

After the inclusion and exclusion criteria were applied, patient demographics (e.g., age at injury, sex, hand dominance) and data on the treatment course (e.g., treatment modality, type of procedure) were extracted from the medical records and the hospital PACS (Picture Archiving and Communication System). All radial head fractures were classified according to Mason–Johnston [21], coronoid fractures according to Regan–Morrey [22] and olecranon fractures according to the Mayo classification [23]. At the time of follow-up, a questionnaire was obtained via mail to assess the OES [24], MEPS [25], Numerical Rating Scale for pain (NRS; 0 = no pain, 10 = worst pain imaginable) [26] and Subjective Elbow Value (SEV) [27,28] to determine the functional outcome. Furthermore, the elbow arc of motion (extension/flexion and pronation/supination) and patient satisfaction (1–6; 1 = most satisfied, 6 = least satisfied) were examined by self-assessment.

### 2.2. Return-to-Sport Metrics

Patients were asked about their participation in sports prior to their elbow dislocation. For those who confirmed participation, the type of sport and weekly duration (hours per week) were documented. Patients were then questioned about their ability to return to sports and categorized as follows:

- Full return to pre-injury sport: Resuming the same sport at the same level as before injury.
- Partial return to pre-injury sport: Returning to the same sport but at a lower level than before injury.
- No return to pre-injury sport but return to other sports: Participating in a different type of sport at any level.
- No return to sports: No participation in any type of sport.

For those who did not return to sports, the reasons were recorded, including limited range of motion, reduced strength, fear of reinjury, and elbow instability. Time to return to sports (in weeks) and weekly duration of participation at follow-up were also assessed. Complications and revision surgeries were documented. An elbow extension/flexion arc of motion less than 100° was considered a complication. Additionally, patients were asked to report their ability and timing of return to work.

### 2.3. Statistical Analysis

The data were analyzed using PRISM version 9.3.0 (GraphPad, San Diego, CA, USA). Before each analysis, normality and homoscedasticity data were tested using the Shapiro–Wilk and Levene’s tests, respectively. Depending on these results, a parametric or non-parametric test (Mann–Whitney U., independent *t* test) was performed. Bivariate data were analyzed using the Fisher exact test. Continuous variables are presented as a mean with a 95% confidence interval (95% CI), ordinal variables as a median with range, and categorical variables as numbers with frequencies and percent. Comparisons of PROMs between patients with and without complications were performed using the Mann–Whitney U test. Missing data were rare (<5%) and handled by complete-case analysis; no data imputation was performed. Significance was determined with *p* < 0.05.

## 3. Results

### 3.1. Demographics

Of 203 patients with complex dislocations of the elbow between 2008 and 2019, a total of 56 patients (26 female/30 male) with a mean age of 49.2 years (range, 17–69) at injury were included after meeting the aforementioned criteria (Figure 1). Demographic data are presented in Table 1.

### 3.2. Injury Pattern, Type of Fracture and Treatment Course

Classification of the fracture type revealed a radial head fracture in 45 (80.3%) patients, a coronoid fracture in 35 (62.5%) patients and an olecranon fracture in 5 (8.9%) patients. Terrible triad injuries were the most common injury pattern, affecting 24 (42.9%) patients. Operative treatment was performed in all cases. Injury pattern, fracture classification and treatment are displayed in Table 2 and Table 3.

Postoperative rehabilitation included immobilization of the elbow for two to three weeks and no weight bearing for 6 weeks. Physiotherapy to passively mobilize the elbow in the extension/flexion plane was started at the second postoperative day. After 6 weeks, active rehabilitation to regain full load bearing was started. Patients who received radial head arthroplasty were permanently limited to 5 kg of weight bearing using the respective arm.

### 3.3. Patient-Reported Outcome Measures (PROMs) and Arc of Motion

Median patient satisfaction at the time of follow-up was high (1, range, 1–5). The mean SEV for the total cohort was 80.2 (95% CI, 74.7–85.8), the MEPS was 83.8 (95% CI, 78.8–88.7) and the OES was 37.8 (95% CI, 35.1–40.5). The median NRS (range, 0–10) was 0 at rest and 1 during load. The mean arc of motion for extension/flexion of the elbow was 115.2° (95% CI, 107.2–123.2°), and the mean arc of motion for pronation/supination was 141.6° (95% CI, 133.2–150.1°). Five patients (8.9%) had an arc of motion of less than 100° for extension/flexion (Table 4).

### 3.4. Return to Sports

Of the 56 patients, 41 (73.2%) were recreational athletes before their injury. Before their injury, 8 (19.5%) patients participated in sports once a week, 26 (63.4%) participated in sports 2–3 times a week and 7 (17.1%) patients participated in sports more than three times a week, with an average time of 4.7 (95% CI: 3.7–5.7) hours a week. Of the 41 patients, 37 (90.2%) patients returned to any kind of sport at a mean of 26.0 (95% CI: 19.7–32.4) weeks after their injury, and 4 (9.8%) patients did not return to any kind of sport. For those who returned to sports, the mean time spent performing sports was 3.7 (95% CI: 2.5–4.8) hours a week. There were 10 (24.4%) patients who did not return to their pre-injury sport, 18 (43.9%) patients who partially returned to their pre-injury sport, and 13 (31.7%) patients who fully returned to their pre-injury sport (Table 5). The reasons the 10 patients were not able to fully return to their sport level were (multiple answers possible) limited strength (70%), fear of injury (70%), limited range of motion (60%), elbow instability (60%) and pain (40%). The most common sports were cycling (63.4%), running (29.3%), swimming (22.0%), and weight training (17.1%).

### 3.5. Return to Work

Return-to-work data were available for 55 out of 56 patients (98.2%). Of those, 6 (10.9%) patients were retired at the time of injury. Of the remaining 49 patients, 40 (81.6%) patients fully returned to work at a mean of 144.7 (95% CI: 94.4–195.0) days, 4 (8.2%) patients partially returned to work and 5 (10.2%) did not return to work at the time of follow-up (Table 5).

### 3.6. Complications

In total, there were 22 complications in 17 (30.4%) patients. The most common complication was elbow stiffness, which occurred in 8 (14.3%) patients, followed by painful hardware in 4 (7.1%) patients, ulnar nerve neuropathy in 3 (5.4%) patients, recurrent dislocation/subluxation in 2 (3.6%) patients, radial nerve neuropathy in 2 (3.6%) patients, ankylosis in 2 (3.6%) patients and non-union of the coronoid process in 1 (1.8%) patient (Table 5).

### 3.7. Revisions

A total of 11 (19.6%) patients underwent revision surgery. The most common revision procedure performed was hardware removal, which was performed in 9 (16.1%) patients, followed by arthrolysis, which was performed in 7 (12.5%) patients. Secondary radial head arthroplasty, application of a hinged external fixator and re-osteosynthesis was performed in 1 (1.8%) patient each.

Patients with complications had a significantly lower MEPS (70.9 [95% CI: 58.3–83.5] vs. 89.4 [95% CI: 85.5–93.2]; *p* = 0.006), lower SEV (67.9 [95% CI: 54.8–81.1] vs. 85.7 [95% CI: 80.7–90.7]; *p* = 0.012) and lower OES (68.8 [95% CI: 55.7–81.9] vs. 84.1 [78.3–90.0]; *p* = 0.043) compared to patients without complications. There were no significant differences between groups in OES (complication: 33.0 [95% CI: 26.7–39.3]; vs. no complication: 39.9 [95% CI: 37.2–42.5] *p* = 0.057).

## 4. Discussion

Overall, the clinical outcome for this study group was favorable. The majority of patients reported a highly satisfactory outcome, and all reported scores (MEPS, OES, SEV) indicated good results. Our reported MEPS of 83.8 points, while slightly lower, is comparable to the values reported in other studies on complex elbow dislocations (MEPS 88.8 and 86.7) [20]. Our complication rate of 30.4% aligns with the 31% rate reported by Gia et al. [4].

The return-to-sport rate was excellent at 90.2%; however, a closer look reveals that only one third of patients were able to fully return to their pre-injury sport. Nearly half (43.9%) returned only partially, and one out of four patients had to switch to a different sport. The overall return-to-sport rate in this study is comparable to that of simple elbow dislocations, which have been reported ranging from 75% to 100% [18,19,29]. For example, in a study investigating judokas, 68% reported returning to their previous performance level, while 22% suffered from a slightly reduced level of performance after conservative therapy for simple elbow dislocations [30]. Müller et al. reported that 90% of patients were able to return to bouldering following a simple elbow dislocation. However, 72% experienced performance limitations due to fear, and only 67% reached or exceeded their pre-injury performance level at mid- to long-term follow-up [31]. Additionally, 15% reported a persistent feeling of instability, while 35% experienced ongoing impairments during sports and daily activities. The study found no significant difference in the time to return to sports between conservative and surgical treatments [31].

In a more general patient population, return to pre-injury level of performance was reported for 56.2% [18] of patients. The most common barriers to full return were limited range of motion, pain, and fear of reinjury [19].

Return to sport is also an important issue in other orthopedic injuries. Only 33.3% of patients with traumatic hip dislocations reported being able to return to their pre-injury level of sports without any limitations, whereas nearly 50% experienced moderate to substantial restrictions in sports participation [32].

High rates of return to sport are reported after ankle fractures, with conservative treatment of stable or non-displaced fractures potentially allowing earlier return. The optimal timing remains unclear due to variability in study quality. For Lisfranc injuries, structured, phase-based rehabilitation is recommended, with full weight bearing at 8–12 weeks and return to sport at 4–6 months. Using this approach, over 90% of elite athletes resume competitive play [33,34].

In this study the most common reasons were limited strength and fear of injury (70%), as well as limited range of motion and elbow instability (60%). This highlights that a rehabilitation program restoring strength as well as functional confidence are important factors next to optimal surgical treatment in securing high return-to-sports rates.

The mean time to return to sport in our study was 26 weeks, which is consistent with the 21.7 to 25.7 weeks reported for simple elbow dislocations [18,19]. Beyond elbow injuries, our results are comparable with those for other common joint injuries like shoulder dislocations and ACL tears. For surgically treated shoulder dislocations, return rates vary but average around 65% returning to their pre-injury level [35]. Likewise, about 80% of athletes with ACL reconstruction return to some sport, but only 65% reach their pre-injury level [29]. The time to return to sport for both of these injuries (4–6 months) is comparable to our findings [36].

In summary, the outcomes for return to sport after complex elbow dislocation are not significantly worse than those for simple elbow dislocations or other common joint injuries, a finding that contradicts our initial hypothesis.

The management of complex elbow dislocations often requires surgical intervention, particularly in cases involving dislocated fractures or significant soft tissue damage. Postoperative rehabilitation plays a critical role in restoring joint function and facilitating a return to pre-injury activities.

Beyond conventional rehabilitation approaches, adjunctive strategies that address both physical and psychological aspects of recovery may further support functional outcomes. Basile demonstrates that music can reduce pain and anxiety, improve mood, and support rehabilitation after traumatic injuries. Although not focused on sport, these effects are relevant for functional recovery. In the context of return to sport after complex elbow dislocation, music-based interventions may enhance rehabilitation adherence, reduce pain-related barriers, and support a more complete return to athletic activity [37].

This study is the first to report on return-to-sport rates following complex elbow dislocation.

Despite its unique contribution, this study has several limitations. The retrospective design and small sample size limit the robustness and generalizability of the findings, although similar constraints are common in studies on complex elbow dislocations. Patient-reported outcomes were assessed at a single follow-up time point, preventing longitudinal analysis. Moreover, the lack of clinical and radiological follow-up limits evaluation of functional recovery and structural healing. The heterogeneity of sports disciplines, including non–upper extremity sports, further reduces generalizability and precludes sport-specific or multivariate analyses. Consequently, larger prospective studies are required to allow for robust multivariate modeling.

Specifically, research should focus on identifying which injury patterns lead to worse return-to-sport rates and whether outcomes vary across different sports, specifically those involving the upper limb.

## 5. Conclusions

This study provides valuable data on return to sports following complex elbow dislocation in non-professional athletes. Complex elbow dislocations remain severe injuries associated with relevant functional limitations and a high complication rate. Nevertheless, good clinical outcomes and high overall return-to-sport and return-to-work rates can be achieved in recreational athletes. While the majority resume sporting activity, almost one out of four patients does not return to their pre-injury sport.

## Figures and Tables

**Table 1:** Overall demographics of 56 patients, with mean values, range and (%).

	Mean (Range; %)
Age at injury, years (range)	49.2 (17.9–69.7)
Sex, n (%)	
Men	30 (53.6)
Women	27 (46.4)
Dominant side injured, n (%)	22 (39.3)
Follow-up, years (range)	6.4 (2.0–12.5)
Workers’ compensation claims, n (%)	21 (37.5)

**Table 2:** Injured structures and injury patterns of 65 patients with n and (%).

		N (%)
Injured anatomic structure	Radial head fracture *	45 (80.3)
	Coronoid fracture	13 (62.5)
	• Reagan/Morrey type I	13 (23.2)
	• Reagan/Morrey type II	15 (26.8)
	• Reagan/Morrey type III	7 (12.5)
	Olecranon fracture ^†^,	5 (8.9)
Injury pattern	Terrible triad injury	24 (42.9)
	Essex-Lopresti lesion	1 (1.8)
	Monteggia-like lesion	5 (8.9)

* all Mason/Johnston type IV. ^†^ all Mayo type III.

**Table 3:** Operative procedures of 65 patients.

Anatomic Structure	Operative Procedure	N (%)
Radial head	ORIF * with screw	11 (16.6)
	ORIF with plate	8 (14.3)
	Resection	3 (5.4)
	Arthroplasty	22 (39.3)
Coronoid	Ante/retrograde screw	7 (12.5)
	ORIF with plate	3 (5.4)
	Suture anchor	12 (21.4)
	Reconstruction with radial head	1 (1.8)
Olecranon	ORIF with plate	3 (5.4)
	ORIF with tension band wiring	2 (3.6)
	Lateral collateral ligament	20 (35.7)
Collateral ligament fixation	Medial collateral ligament	7 (12.5)
	Both collateral ligaments	12 (21.4)

* open reduction, internal fixation (ORIF).

**Table 4:** Patient-reported outcome measures (PROMs) and arc of motion for total cohort. CI: confidence interval, MEPS: Mayo Elbow Performance Score, NRS: Numeric Rating Scale, OES: Oxford Elbow Score, SEV: Subjective Elbow Value with mean (95% CI).

PROM	Mean (95% CI)
MEPS	83.8 (78.8–88.7)
SEV	80.2 (74.7–85.8)
OES	37.8 (35.1–40.5)
NRS rest, median(range)	0 (0–6)
NRS load, median(range)	1 (0–9)
Arc of extension/flexion	115.2 (107.2–123.2)
Arc of pronation/supination	141.6 (133.2–150.1)
Patients with <100° arc of extension/flexion, n (%)	5 (8.9)

**Table 5:** Return to work, sports and complications of recreational athletes with n (%) and 95% confidence interval (CI).

Return to sport (n = 41)	Return to sport, n (%)	37 (90.2)
	Return to preinjury sport level, n (%)	
	• Fully	13 (31.7)
	• Partially	18 (43.9)
Return to work (n = 49)	Fully	40 (81.6)
	Partially	4 (8.2)
	Time to return to work, mean days (95% CI)	144.7 (94.4–195.0)
Complications (n = 56)	Yes, n (%)	17 (30.4)
	Elbow stiffness	8 (14.3)
	Painful hardware	4 (7.1)
	Ulnar nerve neuropathy	3 (5.4)
	Redislocation/subluxation	2 (3.6)
	Ankylosis	2 (3.6)
	Non-union of the coronoid process	1 (1.8)

## Data Availability

Data supporting the results of this study are available upon request to the authors.

## Abbreviations

The following abbreviations are used in this manuscript:

DALY	disability-adjusted life-years
PROM	patient-reported outcome measures
MEPS	Mayo Elbow Performance Score
OES	Oxford Elbow Score
PACS	Picture Archiving and Communication System
NRS	Numerical Rating Scale
SEV	Subjective Elbow Value
CI	confidence interval
